# Object permanence and the development of attention capacity in preterm and term infants: an eye-tracking study

**DOI:** 10.1186/s13052-017-0408-2

**Published:** 2017-10-02

**Authors:** Hokyoung Ryu, Garam Han, Jaeran Choi, Hyun-Kyung Park, Mi Jung Kim, Dong-Hyun Ahn, Hyun Ju Lee

**Affiliations:** 1 Department of Arts & Technology, Hanyang University, Seoul, Korea; 2 Graduate School of Innovation and Technology Management, Hanyang University, Seoul, Korea; 3 Department of Industrial Engineering, Hanyang University, Seoul, Korea; 4 Department of Pediatrics, Hanyang University Seoul Hospital, Hanyang University College of Medicine, 17 Haengdang-dong, Seongdong-gu, Seoul, 133-792 Korea; 5 Department of Rehabilitation, Hanyang University College of Medicine, Seoul, Korea; 6 Department of Psychiatry, Hanyang University College of Medicine, Seoul, Korea; 7 Clinical Research Institute, Hanyang Developmental Medical Center, Seoul, Korea

**Keywords:** Neurodevelopmental outcome, Infant, Premature, Cognition, Eye-tracking

## Abstract

**Background:**

The relationship between premature birth and early cognitive function as measured by eye-tracking data remains unexplored. The aim of this study was to determine the effect of prematurity on the development of object permanence and attention capacity using eye-tracking measures.

**Methods:**

We prospectively studied very low birth weight (VLBW < 1500 g) preterm infants who were admitted to the Neonatal Intensive Care Unit of Hanyang University College of Medicine, Seoul, Korea and visited a follow-up clinic. Using eye-tracking measures, object permanence was assessed in 15 VLBW preterm and 10 term infants at a corrected age of 6–10 months, and attention capacity was measured in 26 VLBW preterm and 18 term children who were age-matched for the corrected age of 6–10 or18 months.

**Results:**

No differences were found in chronologic age (corrected age for prematurity), sex, or maternal education between the study groups. The VLBW preterm infants had lower scores than term infants on eye-tracking measures of object permanence than the term infants did at 6–10 months (*P* = 0.042). The VLBW preterm infants had a shorter referential gaze than the term infants did at 6–10 months (*P* = 0.038); moreover, the length of referential gaze of the VLBW preterm infants was significantly lower at 6–10 months than at 18 months (*P* = 0.047), possibly indicating a delayed trajectory of attention development.

**Conclusion:**

The VLBW preterm infants have different attention capacities and object permanence developmental markers than term infants at the corrected age of 6–10 months.

## Background

With recent advances in neonatology, obstetrics, and neonatal care, the survival rate of preterm infants has significantly increased. Several studies have reported that, despite the absence of structural brain injuries, very low birth weight (VLBW < 1500 g) infants are at greater risk of cognitive impairment involving object permanence, attention deficit, language delay, memory/learning problems and academic performance than term infants [1–3]. Therefore, it is important to provide interventions as early as possible to guide cognitive development [4].

Visual tracking is one of the first basic behaviours to develop attention and communication during cognitive process. Eye-tracking can be used to assess a nonbiased information about gaze direction in response to visual stimuli in infants [5]. Eye tracking research is a good tool to measure visual attention, gaze following, preference, and memory. However, few studies regarding the analysis of eye movement have reported the use of eye tracking in this pre-verbal infant population [6–9]. Since a direct gaze at a specific object during communication involves both referential intention and social interaction, it has been investigated as a marker of cognitive development [8, 10]. In particular, object permanence is a critical cognitive process that requires the ability to pay attention to the object and maintain visual memory, and is considered an early stage of working memory development [11–14]. Eye-tracking technology provides reliable insights into the gaze by assessing the duration of fixation at an area of interest. A few studies have used visual tracking as a predictor of neurodevelopment in infancy, but studies on object permanence and attention in preterm infants during infancy are scarce [15, 16]. The aim of this study was to compare object permanence and attention capacity in response to visual stimuli using eye-tracking in term infants and VLBW preterm infants without major disabilities.

## Methods

### Study population

**Fig. 1:**
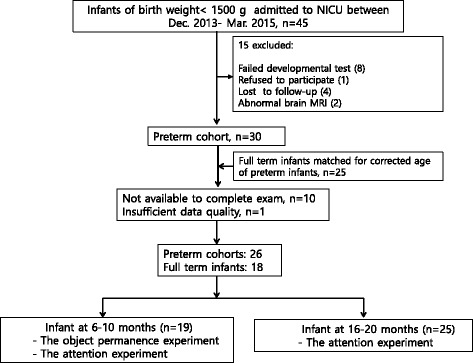
Flow diagram of the study

VLBW infants born at Seoul Hanyang University Hospital in South Korea between December 2013 and March 2015 and admitted to the level 3 Neonatal Intensive Care Unit, were eligible for the study. The inclusion criteria was the VLBW preterm infants who were recruited from the Hanyang Developmental Medical Center for premature infants at the corrected age of 6–10 months or 16–20 months for follow-up because this is the standard practice for neurodevelopmental assessment of VLBW preterm infants [9, 15, 16]. For the control group, we recruited term infants with a gestational age of ≥37 weeks, born at Hanyang University Hospital. These controls were age-matched with VLBW preterm infants for corrected age, and they participated in the study at the well-baby clinic. The exclusion criteria were major congenital malformations, severe brain injury (periventricular leukomalacia on brain magnetic resonance imaging (MRI) or intraventricular haemorrhage ІІІ-ІV grade), metabolic disorder, high risk of developmental delay, retinopathy of prematurity ІІІ-ІV grade or any sign of neonatal encephalopathy or seizure. A total of 45 VLBW preterm infants were recruited from Hanyang Developmental Medical Center for follow-up at the corrected age of 6–10-month or 16–20-month visit. Neurodevelopmental outcomes were assessed in eligible study infants at the 6–10 or 16–20 months visit in the VLBW preterm follow-up program, using the Bayley Infant Neurodevelopmental Screener (BINS), which assesses cognitive capability, language, gross motor skills and fine motor skills. The risk status classifications of BINS is minimally affected by environmental variables, when compared with the Bayley Scales of Infant Development-II, suggesting that it has predictive utility. The age corrected for prematurity was used in the BINS evaluation. Using the BINS score, we categorized the infants in groups at low risk and high risk of developmental delay or neurodevelopmental impairment. The VLBW preterm infants at low risk were eligible for the study. Of the original 45 infants, 8 were high risk based on the BINS evaluation, one parent refused consent, 4 were lost to follow-up and 2 had abnormal brain MRIs. The control group was screened for neurodevelopment at the well-baby clinic at the 6–10 or 16–20 months, using BINS. The control group were born healthy and developed normally, with comparable groups based on the BINS test. Thus, a total of 30 VLBW preterm infants were eligible and 25 full-term infants were matched with the VLBW preterm infants for corrected age to form a control group. However, 10 of the 55 infants were excluded due to incomplete interventions and 1 was excluded due to insufficient data quality. Eye-tracking was finally completed for 26 VLBW preterm infants and 18 term infants (Fig. 1). The object permanence test was assessed in 19 infants at the corrected age of 6–10 months, and attention capacities were compared in 44 infants at corrected ages of 6–10 or 16–20 months in the VLBW preterm cohort, or at these chronologic ages in the term infants. This study was approved by the Hanyang University Institutional Review Board [No. 20141226]. The parents were given a full explanation of the purpose and nature of all procedures, and informed parental consent was obtained before data collection.

### Neonatal risk factors in VLBW preterm infants

Prenatal and neonatal data were based on medical records, including gestational age (GA), birth weight, delivery mode and sex. Small for gestational age, maternal chorioamnionitis, prenatal steroid use, bronchopulmonary dysplasia (BPD, ≥ moderate), retinopathy of prematurity (ROP) and intraventricular haemorrhage I-II were recorded for VLBW preterm infants. Chorioamnionitis was defined by histologic chorioamnionitis or umbilical cord vasculitis of grade 2 or greater, according to the grading system suggested by Salafia et al. [17]. The diagnosis and severity of BPD were determined by assessing the need for supplementary oxygen at 28 days of age and 36 weeks postmenstrual age, the infants breathing air had mild BPD, those who needed <30% supplementary oxygen had moderate BPD, and those needing >30% supplementary oxygen and/or continuous positive airway pressure or a ventilator were defined as having severe BPD [18]. IVH was classified according to Volpe. [19]. Maternal education was categorized as high (more than 10 years), middle (6 to 10 years) or low (less than 6 years), based on the number of years of post-elementary education.

### Eye-tracking assessment

**Fig. 2:**
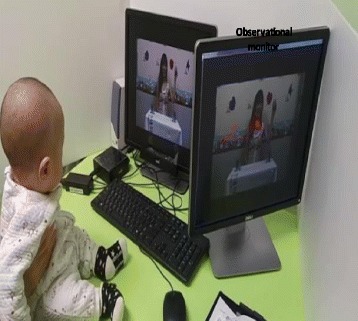
Experimental set-up. Infants were seated on a parent’s lap or on the desk, depending on their height, with their face 60 cm from the display monitor used to show visual stimuli

The experiment was conducted in a assigned room (see Fig. 2), where two 19″ monitors and one eye-tracker (Tobii© X2–60, Tobii, Stockholm, Sweden) were set up. The stimuli for object permanence were presented on one of the monitors (i.e., the stimulus monitor), and the eye-tracker was attached at the bottom of the monitor. The other monitor (i.e., the experimenter monitor) was used by the experimenter to control the experimental session. The infant was seated on the parent’s lap approximately 60 cm from the stimulus monitor. If the infant was not able to maintain this 60 cm distance, he or she was seated on the desk and a parent held him or her from the back (Fig. 2). All eye movements were recorded using the eye-tracking system, which had an accuracy of 0.4 degrees at a rate of 60 Hz. Prior to data collection, a 2-min calibration of the eye-tracking system was carried out. An appropriate sound intensity level (dB) was selected, and the eye-tracking calibration was carried out while the participant was watching an infant-friendly movie. The main experimental session with infants at 6–10 month involved 3 tasks that were counterbalanced and took approximately 10 min.

### Tasks

We designed the experimental tasks to induce attention and object permanence based on previous reports, using a modification of the methods of de Jong et al. and Lowe et al. [1, 9]. The tasks consisted of watching 3 video clips in which an actress presented different stimuli involving 2 cups and a yogurt bottle.

Prior to starting the three tasks, cartoon images of toys with sound effects were used to draw the infants’ attention to the screen. The root mean square (RMS) of the noise of the eye-tracking signals is a measure of data quality. There was no significant difference between the RMS noise in the VLBW preterm and term groups, indicating that the quality of the eye-tracking data was amenable to statistical analysis (Wilk’s Λ = 0.93, F_8,190_ = 1.88, *P* = 0.07).

**Fig. 3:**
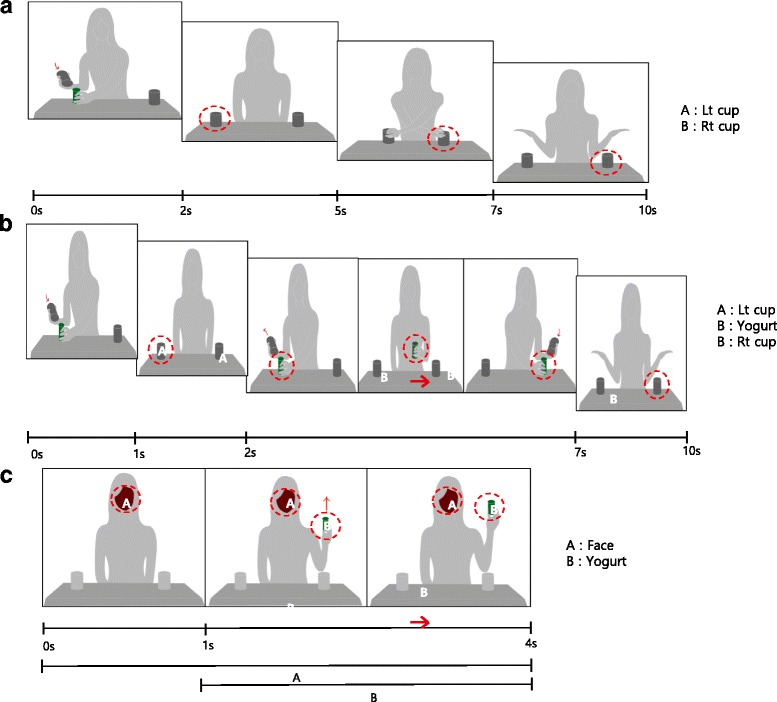
The stimuli for tasks 1, 2 and 3. **a** Task 1: an actress initially hides the yogurt bottle under the table, raises it after 1 s, and then holds it for 3 s **b** Task 2: the actress picks up the yogurt bottle, hides it under the left cup then switches the two cups on the table. She says “*Where is the yummy yogurt bottle?*” Then, starting with the yogurt bottle under the left cup, she exchanges the position of the two cups. **c** Task 3: the actress picks up the yogurt bottle. She initially hides the yogurt bottle under the left cup. A few seconds later, she hides it under the right cup, saying “*I am hiding it again.*” Here, there is no reversal of the two cups, only a change in the location of the yogurt bottle

Task 1: Basic object permanence test (Fig. 3a). The actress picks up the yogurt bottle and hides it under the left cup, then exchanges the two cups on the table. She says, “*Where is the yummy yogurt bottle?*” while hiding the yogurt bottle under the left cup, and then exchanges the position of the two cups again. Here, the infants believe that the left cup and the yogurt bottle are the same object, so following the moving object (i.e., both the left cup and the yogurt bottle) is seen as a basic level of object permanence. Each stimulus was displayed for 10 s. Two stimuli were administered to each child (two trials per task).

Task 2: Advanced object permanence test (Fig. 3b). The actress picks up the yogurt bottle. She initially hides it under the left cup. A few second later, she hides it under the other cup, saying “*I am hiding it again.*” Here, there is no reversal of the two cups, only a change in the location of the yogurt bottle. The infant needs to employ a higher level of cognitive capability to simultaneously manage two hiding steps (i.e., the yogurt bottle under the left cup, and the yogurt bottle under the right cup) and object permanence (i.e., while they observe that the yogurt bottle is moving from the left cup to the right cup) as they retrieve where the hidden object is. Each stimulus was displayed for 10 s. Two stimuli were administered to each child (two trials per task).

Task 3: Attention test (Fig. 3c). The stimulus video shows the actress’s face for 1 s; then she lifts a yogurt bottle to shoulder height and shifts her gaze to the bottle with a verbal indication (“*Let’s look at this. It is a yummy yogurt*”). Gaze direction was not altered in referring to the object. The stimulus is displayed for 4 s. Two stimuli were administered to each child (two trials per task).

### Analysis of the eye-tracking data

For analysis, we developed several areas of interest (AOIs) representing the primary analytic regions where the infants looked and how they responded to the various stimuli. In general, all of the AOIs were rectangular areas that covered the movement of a special object used as the stimulus.

Object permanence test: In Task 1, the left cup was the only AOI. The left cup hiding the yogurt bottle moved to the right position where the right cup was originally located. To analyze the infants’ gaze and object choice followed by basic object permanence, we assessed the infants’ gaze shift at the left cup while the yogurt bottle was hiding, and after moving the left cup. Hence, the infant was considered to have basic object permanence if he or she maintained the gaze on the left cup even after the cup was moved. In Task 2, the stimuli consisted of two types of object permanence: i) the yogurt bottle is hidden under the left cup (then, the actress lifts the left cup and hides the yogurt bottle under the right cup, saying “*I am hiding it again*”), ii) the yogurt bottle is hidden under the right cup. The accuracy of all gaze shifts was scored by (the number of accurate gaze shifts)/(the total number of trials). The maximum object permanence score was 2, and the minimum score was 0.

Attention test: The yogurt bottle was the main AOI, starting from the actress saying “*Let’s look at this. It is a yummy yogurt*.” The distractor was the actress’s face, another AOI. The analysis of Task 3 involved checking how well our participants paid attention to the referential object, the yogurt bottle. To analyze the infants’ referential gaze, we assessed the *looking time* of the target objects by quantifying sustained attention after attention shifted. We calculated the relative proportion of the former (looking time at the yogurt bottle/looking time at both the face and the yogurt bottle). The categorical definition of ‘sustained attention’ was that the infants had to shift their eyes and consistently fix on the target for at least 0.33 s [20].

### Statistical analysis

All the analyses were carried out with SPSS 22 (IBM, Armonk, NY, USA.). We checked data for normal distribution. Data were analyzed for normality of distribution using the Kolmogorov-Smirnov test. Continuous measures were summarized and analyzed using parametric statistics. Normally distributed variables are presented as mean ± SD, and non–normally distributed variables as median value and range. The proportions of looking time, and gaze shift scores, were compared by t-tests or Mann-Whitney U tests in order to identify differences in attention performance and object permanence between VLBW preterm and term infants. A multivariate linear regression analysis was conducted to determine risk factors associated with attention in the VLBW preterm groups. Because looking time during the attention test is a continuous measure, a logistic regression model using the categorical definition of attention was used to compare attention function in VLBW preterm infants with different medical morbidities, while controlling for gestational age, sex, and adjusted age at testing *P* values less than 0.05 were considered statistically significant.

## Results

Object permanence was assessed in 10 VLBW preterm infants and 9 term infants at the age of 6–10 months, and attention capacity was assessed in 26 VLBW preterm and 18 term infants matched for the corrected ages of the VLBW preterm children, at 6–10 or 16–20 months, using eye-tracking measures. The final cohort of VLBW preterm children comprised 26 infants (14 males, 12 females) with a mean birth weight of 989 g and a mean gestational age of 28.9 weeks. The control group comprised 18 full-term infants (13 males and 5 females). Age, sex, adjusted age at testing, maternal age and maternal education did not differ between VLBW preterm infants and term infants (Table 1). Germinal matrix haemorrhage occurred in 15% (*n* = 4) of the 26 infants in the VLBW preterm cohort. A total of 21 infants (81%) was exposed to antenatal steroids, and histologic chorioamnionitis occurred in 11 patients (42%). A total of 8 (31%) had BPD ≥ moderate, but none were dependent on oxygen at the time of assessment. An ROP grade of 1–2 occurred in 13 patients (50%). At matched corrected ages of 6–10 and 16–20 month, there were no statistically significant difference in the total mean BINS scores between the VLBW preterm infants and term infants. The demographic and clinical characteristics of the infants are shown in Table 1.

**Table 1:** Clinical characteristics of the study infants

	Preterm infants (*n* = 26)	Term infants (*n* = 18)	*P*-value
Perinatal characteristic			
Gestational age, wk	28.92 ± 3.89	38.66 ± 1.32	<0.001
Birth weight (g)	989.04 ± 320.77	3108.88 ± 503.29	<0.001
Cesarean section, *n* (%)	21 (80)	11 (61)	0.335
Male gender, *n* (%)	14 (54)	13 (72)	0.183
Small for gestational age, *n* (%)	9 (34)		
Chorioamnionitis, *n* (%)	11 (42)		
Prenatal steroid use, *n* (%)	21 (81)		
BPD ≥ moderate, *n* (%)	8 (31)		
ROP, grade I-II, *n* (%)	13 (50)		
Intraventricular hemorrhage			
Grade I, *n* (%)	4 (15)		
Grade II, *n* (%)	2 (7)		
Socio-demographic characteristics			
Maternal age, years	35.30 ± 3.36	35.44 ± 3.20	0.893
Maternal education, *n* (%)			1.000
High	18 (69)	13 (72)	
Middle	8 (31)	5 (28)	
Low	0	0	
Follow-up characteristic			
6–10 month clinic, months	8.72 ± 1.95 (n = 10)	8.57 ± 1.95 (n = 9)	0.846
16–20 month clinic, months	17.34 ± 2.31 (n = 16)	16.67 ± 1.03 (*n* = 9)	0.619
BINS scores of the patients			
6–10 month	11.20 ± 0.63 (*n* = 10)	11.50 ± 0.75 (n = 9)	0.319
16–20 months	10.75 ± 0 .70 (*n* = 16)	10.66 ± 0.77 (n = 9)	0.811

Data are presented as mean ± SD or number (%)

*BPD* bronchopulmonary dysplasia, *ROP* retinopathy of prematurity

There was no significant difference in basic object permanence capability between the VLBW preterm infants and term infants (0.400 ± 0.699 and 0.222 ± 0.441, respectively, *p* = 0.633). However, the VLBW preterm infants had a significantly lower score on eye-tracking measures for assessing advanced object permanence at 6–10 months (0.400 ± 0.516 vs. 1.111 ± 0.782, *p* = 0.042) (Table 2).

**Table 2:** Differences in object permanence between preterm and term infants

	Preterm infants	Term infants	*P*-value
*N*	10	9	
Basic object performance	0.400 ± 0.699	0.222 ± 0.441	0.633
Advanced object performance	0.400 ± 0.5164	1.111 ± 0.782	0.042

Data are presented as mean ± SD or number

**Fig. 4:**
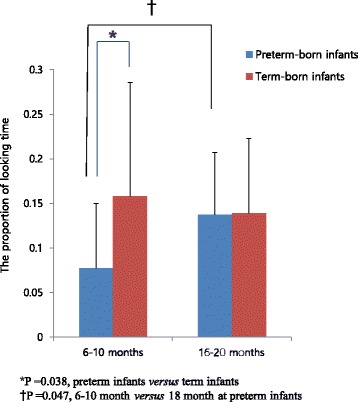
The proportion of time spent gazing at a reference was significantly shorter in the preterm infants than in the term infants at 6–10 months

The proportion of looking time for the referential gaze was significantly lower in the VLBW preterm infants than in the term infants at 6–10 months (0.077 ± 0.073 vs. 0.158 ± 0.128, *p* = 0.038). Term infants spent significantly longer looking at the referential object than the preterm infants at 6–10 months, but this referential gaze was comparable between 16 and 20 months old groups of VLBW preterm infants and controls. The proportion of looking time for the referential gaze in the VLBW preterm infants was also significantly lower at 6–10 months than at 16–20 months (0.077 ± 0.073 vs. 0.137 ± 0.070, *p* = 0.047) (Fig. 4).

There were no significant differences in looking time within 0.33 s, in the attention performance for target fixation in infants after controlling for gestational age, sex, and adjusted age at testing (data not shown).

## Discussion

We compared eye-tracking data for object permanence at a corrected age of 6–10 months in low-risk VLBW preterm infants with data for term infants at age 6–10 months. The VLBW preterm infants exhibited significantly lower object permanence, suggesting that even “healthy” preterm infants follow a delayed cognitive processes than term infants. Prospective and follow-up studies have shown that VLBW preterm infants are at risk of cognitive dysfunction [3]. In the development of cognitive functioning, object permanence may be an early diagnostic marker of neurodevelopment and a critical item for assessing early working memory capacity in preterm children. Object permanence mediates the ability to both pay selective attention to information and inhibit interfering information. Lowe et al. [9] showed that higher object permanence scores were significantly related to higher cognitive and language scores on the Bayley Scales-III at 18–22 months of age in preterm infants, after controlling for socio-economic status and preterm morbidities.

Using an eye-tracking system, we compared VLBW preterm infants and term infants with respect to gaze, that is, time spent looking at a target object when they shifted their attention in the direction of the referent. Term infants spent significantly longer looking at the referential object than the VLBW preterm infants. Furthermore, referential gaze representing shared attention elicited a significantly longer looking time in the term infants than the VLBW preterm infants at 6–10 months of age, while in the VLBW preterm infants referential gaze and looking time were longer at 16–20 months than at 6–10 months of corrected age.

These findings point to a different developmental trajectory of the orienting attention system in VLBW preterm infants and, in line with previous studies, suggest that VLBW preterm infants are predisposed to language impairment and attention deficit/hyperactivity disorder later in life despite having no major disabilities [2, 10, 21]. Follow-up neurodevelopmental assessments and screening in children born prematurely ought to be encouraged, not only to decrease the deleterious consequences of prematurity, but also to achieve optimal development. Recent studies have focused on cognition and executive functioning in children born VLBW preterm infants [22–24]. For example, Johnson et al. [25] suggested that newborn infants have an innate preference for looking at face-like stimuli, with specific attention given to the eye region very soon after birth through a process of subcortical face processing. Gaze- following occurs between 6 and 12 months of age, while referential gaze is a more advanced cognitive achievement that emerges between 12 and 18 months [7]. However, the age at which infants begin to engage in referential gaze is related with individual differences in this skill between 6 and 18 months [20, 26]. Morales et al. [27] reported that infants as young as 6 months start to respond to referential gaze, which was related to subsequent language development. In the present study, referential gaze with looking time on the referent target was measured as early as 6 months, before they fully develop subsequent performance of joint attention. There is considerable evidence to support the idea that referential gaze is critical for the later development of communication in infants [6, 28, 29]. In the present study, referential gaze as a milestone of the development of social cognition was less advanced in VLBW preterm infants than term infants even at the early ages of 6–10 months and 16–20 months.

Telford et al. [16] demonstrated that VLBW preterm infants have shorter attention spans in response to social stimuli of increasing complexity than healthy term controls at a median age of 7 months, pointing to atypical attentional control. De Jong et al. [1] studied the development of the attention capacity of 123 VLBW preterm infants at 18 months using eye-tracking, and compared it to that of 101 term children. The VLBW preterm infants had lower orienting and alerting attention abilities at 18 months, suggesting that they are at increased risk of attention problems at school age. However, our findings differ from those of several other studies that suggested that early visual experience after preterm birth accelerated infants’ visual and attentional development by speeding up maturational processes [30, 31]. Future research should address whether increased visual activity in response to additional visual stimuli in the extrauterine environment can affect early and/or later maturational processes. The present study is tested with widely used standard range of age groups from infants at 6–10 months or 16–20 months, which may not be an exact indicator of the extent of children’s performance with variations. However, we assessed eye-tracking data based on previous studies that have also analyzed neurodevelopmental outcome from preterm infants in those age span [9, 15, 16].

Whether the cognitive differences seen in preterm infants were the result of an altered brain microstructure or disrupted cortical network during brain development is unclear. Different aspects of early visual function mature at different times and are probably related to different underlying subcortical and cortical mechanisms. There are a few reasons why preterm infants and term infants might shift gaze to a particular object differently. Previous studies demonstrated that preterm children with intraventricular hemorrhage І-ІІ and BPD tended to have lower object permanence scores and shorter attention span [9, 32, 33]. Pel et al. [34] suggested that children born extremely preterm may have delays in response times to specific visual properties in processing visual information, suggesting deficits in neuronal connectivity in visual pathways at a microstructural level. The visual processing problem related to preterm birth might also influence our eye-tracking result, even though these infants had no ophthalmological impairments or structural brain damage on conventional MRI. None of the VLBW preterm infants in the present study had intraventricular hemorrhage ІІІ-ІV or periventricular leukomalacia, but 8 of the 26 preterm infants had moderate to severe BPD. However, logistic regression analysis revealed that the VLBW preterm infants’ attention performance was not associated with neonatal factors in clinically stable VLBW preterm infants due to a large number of covariates and small number of groups. Our data were not collected with a longitudinal design and the number of infants in each subgroup subdivided by postmenstrual age was too small to be amenable to statistical analysis. Additional studies with a larger cohort would help to better define early visual functioning in VLBW preterm infants.

## Conclusions

The result of our study lead us to consider that healthy VLBW preterm infants might have additional risks of later problems in social and attentional areas. We suggest that a structured assessment of cognitive functioning could be added to clinical practice to detect early deficits in object permanence and referential gaze although these children develop normally in the early years.
